# Magneto-elasto-electroporation (MEEP): *In-vitro* visualization and numerical characteristics

**DOI:** 10.1038/srep32019

**Published:** 2016-08-26

**Authors:** Soutik Betal, Binita Shrestha, Moumita Dutta, Luiz F. Cotica, Edward Khachatryan, Kelly Nash, Liang Tang, Amar S. Bhalla, Ruyan Guo

**Affiliations:** 1 Department of Electrical and Computer Engineering, University of Texas at San Antonio, San Antonio, TX 78249, USA; 2 Department of Biomedical Engineering, University of Texas at San Antonio, San Antonio, TX 78249, USA; 3 Department of Physics, State University of Maringá, Maringá, PR – 87020-900, Brazil; 4 Department of Physics and Astronomy, University of Texas at San Antonio, San Antonio, TX 78249, USA

## Abstract

A magnetically controlled elastically driven electroporation phenomenon, or magneto-elasto-electroporation (MEEP), is discovered while studying the interactions between core-shell magnetoelectric nanoparticles (CSMEN) and biological cells in the presence of an a.c. magnetic field. In this paper we report the effect of MEEP observed via a series of *in-vitro* experiments using core (CoFe_2_O_4_)-shell (BaTiO_3_) structured magnetoelectric nanoparticles and human epithelial cells (HEP2). The cell electroporation phenomenon and its correlation with the magnetic field modulated CSMEN are described in detail. The potential application of CSMEN in electroporation is confirmed by analyzing crystallographic phases, multiferroic properties of the fabricated CSMEN, influences of d.c. and a.c. magnetic fields on the CSMEN and cytotoxicity tests. The mathematical formalism to quantitatively describe the phenomena is also reported. The reported findings provide insights into the underlying MEEP mechanism and demonstrate the utility of CSMEN as an electric pulse-generating nano-probe in electroporation experiments with a potential application toward accurate and efficient targeted cell permeation.

Electroporation in brief is the universal phenomenon of opening conductive pores on cell membrane when exposed to a millisecond to nanosecond electric pulse with tens of kV/cm intensity applied from a few microseconds to millisecond time scale at a sub-millimeter to micrometer distance^1^,^2^,^3^,^4^,^5^,^6^. This process has a broad range of applications, including loading electroporated cells with small molecules^7^,^8^ (e.g. fluorescent tracers and anti-cancer drugs) and large molecules (e.g. long chain amino acids, proteins or DNA) and has been successfully employed for over a decade for DNA or gene transfection, protein insertion, cell fusion and enhanced uptake of metallic nanoparticles for improved drug delivery^4^,^9^,^10^. Based on research conducted in the last few decades on the physical mechanism of electroporation, a broad yet phenomenological knowledge of electroporation has been established. This knowledge has been extensively influenced by experiments based on studies on electro-permeabilization of the phospholipids bilayer membrane. In these experiments, change in the membrane permeability/conductivity and mechanical rupture, along with a cell’s impedance measurements while exposed to electric pulses have been studied. The uptake of fluorescent molecule enzymes and antibodies by cells and imaging of a cell’s transmembrane potential while being exposed to an electric pulse has also been studied^11^,^12^,^13^,^14^,^15^,^16^. Recent studies on the effect of calcium influx on intracellular transport and membrane repair after exposure of cells to a pulsed electric field yielded visible confirmations of the electrical interactions between negative electrodes and cations inside cells while opening nanopores on a cell membrane^17^,^18^,^19^. Most of the electroporation experiments were carried out on (i) a suspended cell–patch model^5^, (ii) a single cell in a microfluidic chamber^20^,^21^, or (iii) the planar bilayer lipid membrane^22^,^23^. These experiments have been conducted using instruments such as an electroporation cuvette/tungsten nano-electrodes connected to a strong electric pulse generator, e.g., Gene Pulser MXcell electroporation system, which exposes cells to a pulsating electric field and allows researchers to study the characteristic changes in a cell by specifically analyzing opened pores on phospholipids bilayer membrane.

Electroporation primarily results in a transmembrane voltage change, which leads to a transient change in the semi-permeability of the cell membrane. The specific conductance of a cell membrane is usually smaller than 10^−3^ S-cm^−2^ under normal physiological conditions. Significant increases in the membrane specific conductance up to 1 S-cm^−2^ within microseconds of exposure to electrical pulses results in the rearrangement of the phospholipids bilayer^24^,^25^. An individual cell membrane is basically a self-assembled 5 nm phospholipids bilayer formed in an aqueous medium due to the hydrophobic effect. The center of the phospholipid bilayer sheet is a hydrophobic zone^22^. Several electroporation experiments have verified that nanopores open near the negative electrodes that are used to apply the pulsed electric field. The negative electric field lines induce an electrostatic repulsion on the negative charge phosphate heads of the phospholipids bilayer arrangement, which results in a displacement of the phosphate heads against and away from the field lines. The phospholipid cannot flip flop the hydrophilic tail in a polar/aqueous region which leads to the creation of volcanic-shaped aqueous pores^23^,^24^,^25^,^26^,^27^. This dislocation of phospholipids on the cell membrane results in a transmembrane voltage change; a finite permeability of the phospholipids bilayer allows current to flow through, inducing a phase transition of the phospholipid bilayer. Both of these events give rise to conformational changes in the cell membrane thereby increasing membrane permeability to ions, molecules and macromolecules. In 1990, Chang and Reese reported visual confirmations of the formation of various volcano-shaped pore openings of diameters varying from 20–120 nm within 40 ms of an applied RF electric field^28^. In their experiments, Rae and Levis also verified single cell electroporation by studying one volcanic-shaped nanopore on a cell membrane^29^. Chang and Reese as well as several other researchers, have also reported the closing of these nanopores after ~10 sec of electric field exposure when kept in an electrolyte, which clearly demonstrates that the hydrophobic tails of the phospholipids in the bilayer membrane tend to cluster again in a polar medium due to the hydrophobic effect and close the nanopore. Although the process of electroporation is a reversible process, i.e., pores open and close on the cell membranes after electric pulse exposure, controlling the opening of a single nanopore and analyzing a biological cell, especially live cells, are very tedious and nearly impossible. One of the reasons for this complexity is the lack of nano-probes that can apply localized electric pulses to open a controlled single nanopore. Because the voltage applied is not localized, a large area of the cell membrane is exposed to the electric pulse, and the number of opened nanopores of variable sizes on the membrane will depend on the exposure time and the angle of the voltage application. Due to this uncontrolled applied electric potential, most of the time, cell death or rupture occurs, and the electroporation study fails. To overcome this complexity of single nanopore study on cell membrane, we propose a new nano-technological approach.

Our present study is primarily focused on exploring the mechanism of Magneto-elasto-electroporation (MEEP). MEEP is the phenomenon of a nanopore opening on a cell membrane due to its interaction with core-shell magnetoelectric nanoparticles under the influence of an alternating current (a.c.) magnetic field. The core-shell magnetoelectric nanoparticle (CSMEN) investigated comprises a magnetostricitve core of cobalt ferrite (CoFe_2_O_4_) and a ferroelectric shell of barium titanate (BaTiO_3_) to achieve MEEP across cell membranes. The core of the CSMEN is encapsulated by a piezoelectric shell. The encapsulated core is capable of producing a magnetoelastic wave emission under the influence of an a.c. magnetic field. The core of the CSMEN experiences strain in the form of directional shape deformation in the presence of an a.c. magnetic field. The strain on the CSMEN core will generate a magnetoelastic wave that is absorbed by the shell as a pressure wave. The absorbance of the pressure wave by the BaTiO_3_ shell changes the surface potential due to the shell’s piezoelectric property. When placed within a sub-micrometer distance of a cell membrane, the continuous change of the surface potential of CSMEN under the influence of an a.c. magnetic field results in a cell’s transmembrane voltage change across the bilayer phospholipid membrane. Under the influence of the a.c. magnetic field, the CSMEN emits a pulsed negative localized surface potential with a frequency that varies in accordance with/response to the exciting frequency variations. The bilayer phospholipid membrane has a negative phosphate head that experiences repulsion due to the CSMEN pulsed localized surface potential. The repulsion primarily results in phospholipid position dislocation. The whole area of the phospholipid membrane experiencing the electrical pulses from the CSMEN will undergo dislocation of phospholipids in the hydrophilic region to attain the lowest energy resulting in volcanic-shaped nanopores on the cell membrane. The CSMENs due to the magnetic moment will further penetrate the membrane through these electrically opened nanopores toward magnets. Furthermore, due to the pore opening, non-polar fatty acid again comes in contact with the polar or hydrophilic region and the pore starts to close within a few seconds due to the hydrophobic effect, as discussed earlier. An illustration of the MEEP mechanisms is presented in Fig. 1, where Fig. 1(a–c) show the cell permeation by the CSMEN, where electromechanical coupling of the multiferroics properties of CSMEN under the influence of an a.c. magnetic field results in the nanopore opening and CSMEN’s penetration through the bilayer membrane into the cell. Fig. 1(d–f) show the schematics of the process of opening the volcanic-shaped nanopore on the cell membrane due to the negative phosphate head being displaced from its original position after electrostatic repulsion from a strong, localized pulsing negative electric field produced by the CSMEN under the influence of an a.c. magnetic field.

This study focused on the magneto-elasto-electroporation (MEEP) effect, where we manipulated targeted electroporation in HEP2 cells by causing them to interact with core-shell magnetoelectric nano particles. We fabricated and characterized the core (CoFe_2_O_4_) – shell (BaTiO_3_) magnetoelectric nanoparticles (CSMEN) used in this study. A series of experiments were carefully designed and conducted to examine the following issues: (A) crystallographic phases and multiferroic properties of the CSMEN, (B) influences of d.c. and a.c. magnetic fields on the CSMEN as functions of their amplitudes and frequencies; (C) the cell electroporation phenomenon and its correlation with the magnetic field modulated CSMEN. The experiments and the analysis reported in this paper demonstrate that the CSMENs retained the physical, electrochemical, magnetic, and piezoelectric properties associated with their respective core and shell components based on the corresponding diffraction patterns, zeta potential values, magnetic hysteresis loops, and piezoelectric force microscopic responses. Furthermore, the core-shell nano-composite structure studied enables novel multiferroic couplings between the magnetostrictive properties of the cobalt ferrite (CFO) core and the piezoelectric properties of the barium titanate (BT) shell, which results in modulation of the surface potential of the CSMENs directly by the externally applied a.c. magnetic field. The combination of Lorentz force and time-dependent surface potential change, as hypothesized and verified in this work, gives rise to the directional movement of the CSMEN and the targeted electroporation of biological cells.

## Results

### Crystallographic Phases and Multiferroic Properties of the CSMEN

#### Synthesis of BaTiO_3_-Coated CoFe_2_O_4_ Nanoparticles

The Core-Shell Magnetoelectric (CSMEN) nanoparticle composites were synthesized using hydrothermal methods. The CoFe_2_O_4_ nanoparticles used were obtained from a commercial source (Alfa Aesar Inc.). Barium carbonate (BaCO_3_) and titanium iso-propoxide (Ti(OCH(CH_3_)_2_)_4_) were mixed with citric acid in separate containers to obtain the Ba and Ti citrate solutions. To form a uniform coating of BaTiO_3_ on the CoFe_2_O_4_ nanoparticles, these citrates were then mixed with CoFe_2_O_4_ nanoparticles in ethylene glycol and heated at 100 °C to pyrolyze the solution. To stabilize the barium titanate shell and achieve the intended crystalline characteristics, the mixture was dried and heated further at 800 °C for 8 hours in a low supply of oxygen to prevent oxidation of the ferromagnetic nanoparticles. Finally, the dried powder was washed several times using ethanol and de-ionized water and sonicated in an ultrasonic bath to obtain the final crystallized composites of BaTiO_3_-coated CoFe_2_O_4_ nanoparticles.

#### Microstructure of the Nanoparticles

**Figure 2:**
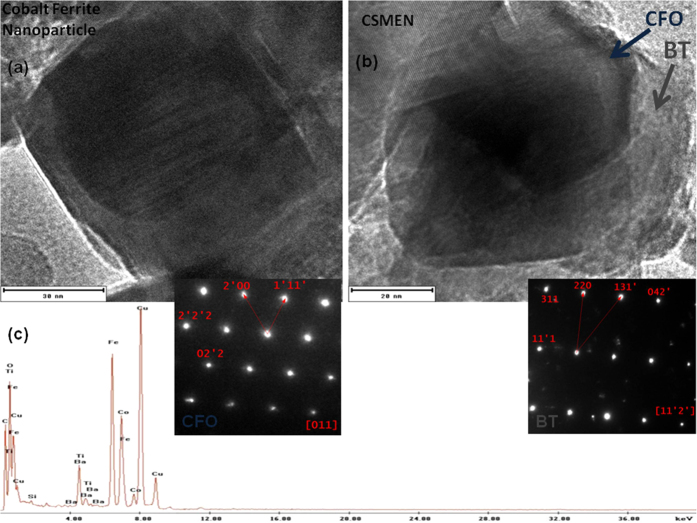
Morphology and microstructure of the synthesized CSMENs demonstrated by Transmission Electron Microscopy image and the selective area diffraction patterns insets (**a**) for the CFO core and; (**b**) for the CSMEN’s BT shell; (**c**) The energy dispersive spectrum for the composition analysis of the CSMEN.

The microstructure of the synthesized CSMENs was studied via transmission electron microscopy (JEOL 2010F), as shown in Fig. 2. The CoFe_2_O_4_ nanoparticles were uniform in shape and size with predominant cubic morphology. The size of the BaTiO_3_-coated CoFe_2_O_4_ nanoparticles was estimated, based on TEM images (Fig. 2(a,b)), to be ~80 nm, while the CoFe_2_O_4_ core measured ~50 nm. The measured size of cobalt ferrite nanoparticles was consistent with the manufacture’s specification, proving that we were observing the cobalt ferrite core in CSMEN. To substantiate the crystalline nature of the CSMEN, selected area electron diffraction (SAED) measurements were carried out- for the core and the shell areas. Gatan Digital Micrograph 1.85 and JEMS were used to index the diffraction pattern and to calculate the zone axis. The diffraction patterns shown as inserts of Fig. 2(a,b) demonstrate the <011> zone axis for CFO and <1
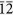
> zone axis for BT, substantiating the single crystalline nature of the CFO and BT correspondingly. Semi-quantitative energy dispersive X-ray analysis (EDXA) was performed to establish the chemical identity of the particle. As shown in Fig. 2(c), the energy peaks attributed to Ba^2+^, Ti^4+^, Co^2+^, Fe^3+^and O^2−^ were quite prominent. Visible peaks of Copper and Carbon in Fig. 2(c) were associated with the grid used for the measurement.

#### Size Analysis

**Figure 3:**
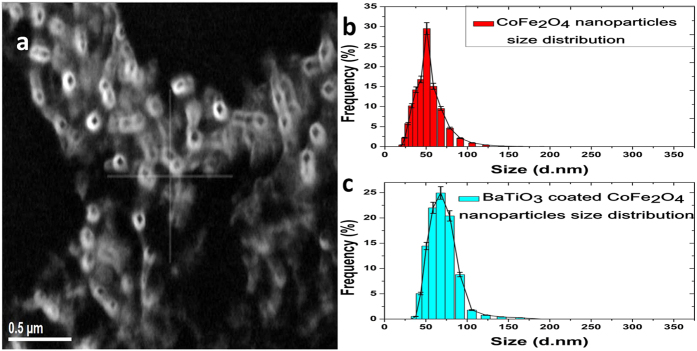
Cobalt ferrite nanoparticles and CSMEN size Analysis. **(a)** Atomic force Microscopy (AFM) image-Coated Nanoparticles i.e. CSMENs; **(b)** Meta-ZetaSizer – measurement curve of size analysis of cobalt ferrite nanoparticles; **(c)** Measurement data of size measurement of BaTiO3 coated Cobalt Ferrite nanoparticles (CSMEN).

For the size analysis of CSMEN, measurements were made using the dynamic light scattering (DLS) method. Cobalt ferrite and barium titanate-coated CSMENs were mixed in de-ionized water, and the mixture was placed in an ultrasonic bath for 12 hours. The solution was analyzed using a Zetasizer Nano ZS (Malvern Instruments Ltd.). Figure 3(b) shows the DLS size measurement of CFO nanoparticles; Fig. 3(c) shows the DLS size measurement of BaTiO_3_-coated Cobalt Ferrite nanoparticles at a molar mass composition ratio of 40:60. The cobalt ferrite nanoparticle’s size was measured as being 50.09 ± 2.5 nm, while the CSMEN size was 78.8 ± 3.25 nm. AFM measurements also confirmed the size in the same range. The AFM results and related sample preparation are discussed in the supplemental material (Fig. S1). Both the scanning and 3-D topography image of the CFO nanoparticles and CSMEN on a positively charged atomically smooth mica surface showed that the CFO nanoparticles had a size of approximately 50–55 nm. CSMEN size was approx. 75–80 nm. The AFM microscopic image in Fig 3(a) shows the core-shell magnetoelectric nanoparticles with barium titanate coating on cobalt ferrite nanoparticles. The AFM data, TEM size analysis and Meta zeta-sizer measurement data appear to be in good agreement.

#### Zeta Potential Measurements

**Figure 4:**
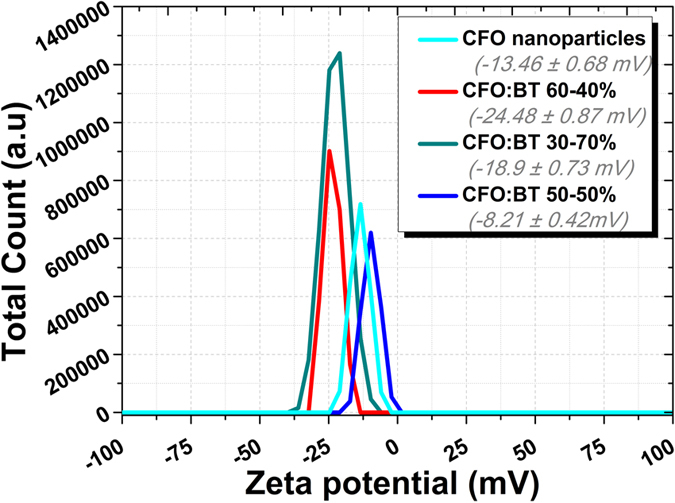
Zeta-potentiometer measurements. Zeta potential measured using malvern zeta-potentiometer for CoFe_2_O_4_ nanoparticles and BaTiO_3_ coated CoFe_2_O_4_ nanoparticles (40:60 molar mass ratios), (30:70 molar mass ratios) and (50:50 molar mass ratios).

Zeta potential measurements were performed using the Zetasizer Nano ZS and a Disposable Capillary Cell (DTS1070). The magnitude of the zeta potential indicates the degree of electrostatic repulsion between adjacent, similarly charged particles in dispersion^30^,^31^. The zeta potential measurement illustrates that the surface potential of the nanoparticles is highly sensitive to the BaTiO_3_ coating. Figure 4 shows the zeta potential of the cobalt ferrite nanoparticles and barium titanate-coated cobalt ferrite nanoparticles, with different molar mass percentage ratios of CFO:BT: as 70:30%, 60:40% and 50:50%. The Zeta-potentiometer results show that the CFO nanoparticles possess negative zeta-potential that changes after the BaTiO_3_ coating. The change in zeta potential is not linearly dependent on the BT coating ratio; rather there is an optimal composition, i.e., CSMEN with 60% cobalt ferrite and 40% barium titanate has the highest recorded zeta potential value among the three compositions prepared.

#### PFM Studies: Single Crystalline State of BaTiO_3_ Shell

**Figure 5:**
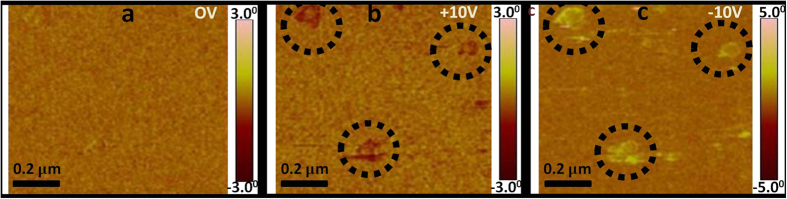
Piezo Response Force Microscopy (PFM) measurements. **(a)** No phase difference observed on CSMEN while applying 0 V on PFM tip; **(b)** The max phase difference on CSMEN observed while applied tip voltage of +10 V was −3° whereas; **(c)** The max phase difference on CSMEN observed while applied tip voltage of −10 V was +5°

PFM samples were prepared as discussed in the Method section and described in ref. 32. The experimental configuration used for the Piezo-response Force measurements was described in ref. 33. The Piezo-response Force Microscopy measurements were performed with a tip voltage of 0 V and at a tip biased voltage of +10 V and −10 V. The PFM results in Fig. 5, clearly show the polarization state switching, signified by the phase difference of the individual CSMEN due to the converse-piezoelectric effect, where in Fig. 5(a), it can be observed that no phase difference of CSMEN was observed while applying 0 V. Figure 5(b) shows the maximum phase difference (−3°) of the CSMEN observed for an applied tip voltage of +10 V whereas, in Fig. 5(c), the maximum phase difference of the CSMEN observed for an applied tip voltage of −10 V was +5°. This result provides clear evidence that the BaTiO_3_ coating is ferroelectric in nature and underlies its tetragonal perovskite symmetry at room temperature. Moreover barium titanate polarization states may be fully controlled by the electrostatic potential at the core-shell interface, and no poling is required to attain magnetoelectricity in the CSMEN. The strain of the CFO core may be transferred to the BT shell and results in a surface potential change on the BT coating. A dynamic transfer of a mechanical wave from core to shell in the CSMEN is also confirmed through opto-acoustic measurements as discussed in a later section.

#### Magnetic Hysteresis Behavior

**Figure 6:**
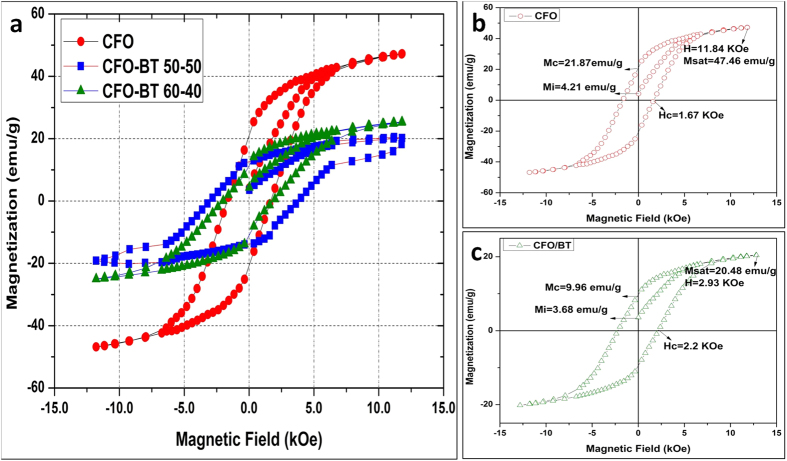
Ferromagnetic hysteresis Curve. **(a)** The hysteresis measurement results show the ferromagnetic behavior of cobalt ferrite nanoparticles with peak magnetization of 51 emu/g whereas after coating with different amount of Barium titanate the peak magnetization decreases to 22 emu/g with 60% CFO-40%BT and 18.4 emu/g with 50%CFO-50%BT; **(b)** Hysteresis measurements of cobalt ferrite nanoparticles and; **(c)** Hysteresis measurements of CSMEN with 60% CFO-40%BT composition.

Magnetic hysteresis curves measurements were carried out to study the change in magnetization of cobalt ferrite nanoparticles after coating them with barium titanate. Nanoparticles were placed in a small Teflon cuvette in a vibrating sample magnetometer mounted between the pole pieces of a biased d.c. electromagnet (GWM-3473); measurements were obtained using a highly sensitive gaussmeter (Lakeshore-425) and a Hall probe. As shown in Fig. 6(a), the hysteresis curve demonstrates the non-linear ferromagnetic behavior of the cobalt ferrite nanoparticles with changes in the magnetic field intensity. It can also be seen that the cobalt ferrite nanoparticles have a saturation magnetization of 51 emu/g. After they are coated with a different composition of barium titanate, the saturation magnetization decreases to 22 emu/g with 60% CFO-40%BT and 18.4 emu/g with 50%CFO-50%BT. This decrease in magnetization clearly indicates that the barium titanate layer is coated on the cobalt ferrite nanoparticles and is reducing the intensity of magnetization in the CFO nanoparticles. A ferromagnetic hysteresis loop was observed while particle magnetization response was being recorded using a highly sensitive magnetometer by applying a biased magnetic field. Figure 6(b), shows the non-linear ferromagnetic behavior of cobalt ferrite nanoparticles; Fig. 6(c), shows ferromagnetic behavior of CSMEN. As shown in Fig. 6(b), in case of the cobalt ferrite nanoparticles, the saturation magnetization M_sat_ was found to be 47.46 emu/g under a magnetic field of 11.84 KOe with the Coercive field as 1.67 KOe and the remnant magnetization measured as 21.87 emu/g. On the other hand, when the CSMENs were analyzed, M_sat_ was observed as being 20.48 emu/g under a magnetic field of 12.93 Koe, with the coercive field at 2.2 KOe and the remnant magnetization measured as 9.96 emu/g (can be seen in Fig. 6(c)). As observed whereas the coercive field and saturation magnetic field seem to increase, the remnant magnetization and saturation magnetization seem to decrease when cobalt ferrite nanoparticles are coated with barium titanate.

Furthermore analysis of the magnetic measurements was done by using Magnetic Force Microscopy (MFM) measurement and is discussed in supplemental material (Fig. S2). MFM was performed to analyze the magnetic domains of the particle in a nanoscale region. For MFM standard pyramidal shaped etched silicon tips of MESP type supplied by Bruker with tip radius being around 35 nm has been used. Magnetic contrast it captures is attributed to its cobalt-chromium coating, which has a coercivity of ~400 Oe and a magnetic moment of 10^−13^ emu. In order to ensure a predominant orientation of the magnetic vector field along the major probe axis, the probe was magnetized along the cantilever using the permanent magnet suppled by Digital Instruments company prior to taking MFM measurements. MFM has been executed in lift off mode where during the lift off mode the magnetized tip was lifted up by 100 nm, i.e. higher than the size of the nano particles to eliminate any kind of topographical interaction and the deflection of the tip caused purely by magtetic attraction or repulsion between the probe and the particle construed the amplitude and phase images over an area of 100 nm × 100 nm depicting the presence of magnetic domains as shown in supplemental material (Fig. S2). The amplitude and the phase images represent the amount of shift brought about to the intial amplitude and phase to which the tip was tuned to, by the magnetic interaction between the magnetic domains of the particle with that of the magnetized tip. While the darker regions signify attraction, the brighter regions shows repulsion experienced by the MFM magnetized tip due to magnetization of the nanoparticle.

#### Opto-acoustic measurements

**Figure 7:**
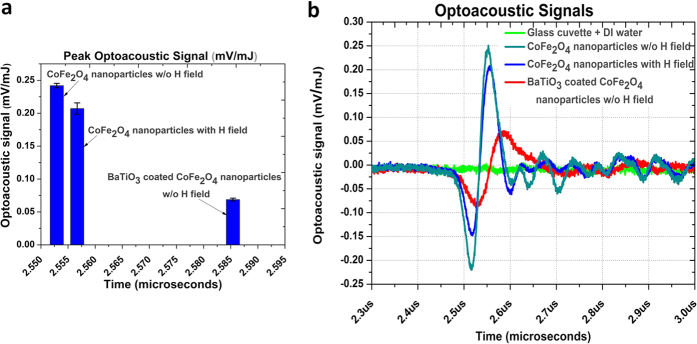
Opto-acoustic Emission data and response curve. **(a)** Photoacoustic emission peak intensity of cobalt ferrite nanoparticles decreases when an a.c. magnetic field is applied and further when CSMEN were analyzed w/o an a.c. magnetic field application, the OA intensity peak further reduces; (**b**) Opto-acoustic response curves of reference, CFO nanoparticles and CSMEN are compared.

The influences of d.c. and a.c. magnetic fields on the CSMEN as functions of amplitude and frequency are further studied via opto-acoustic measurements. Using an all optical opto-acoustic approach as described in earlier reports^34^,^35^,^36^. Pure cobalt ferrite nanoparticles were placed in a glass cuvette that was then filled with liquid (de-ionized water) until reaching the top of the cuvette (~4 ml). Because the cobalt ferrite nanoparticle’s density is greater than that of water (1 g cm^−3^), cobalt ferrite nanoparticles were found at the bottom of the cuvette forming a thin bed. An optical parametric oscillator (OPO) (EKSPLA model 342NT) laser system pumped by a Nd:YAG pulsed laser at 355 nm was tuned to 520 nm and used as an excitation source with a pulse duration of 3.6 ns and a repetition rate of 10 Hz, each pulse having a top hat profile. The energy of the laser was monitored upon focusing this pulsed energy to a fluence of ~2 J cm^−2^ during the duration of the experiment, forming the maximum intensity for any given sample, and was kept constant at ~23 ± 1 mJ pulse^−1^ upon the exposure to the pulsed nanosecond Nd-YAG laser. Upon pulsed excitation, a thermal expansion was produced as a result of light absorption by the nanoparticles, which in turn emitted a pressure (acoustic) wave capable of travelling through an acoustically coupled medium such as water. To measure this acoustic wave, a 5 mW HeNe-laser probe beam was passed through the water, just above the nanoparticles. The resulting elastic wave transiently changed the refractive index of the water deflecting the probe beam from its original optical path. The deflection was measured by a sensitive four-quadrant position sensitive detector and the response was recorded using an oscilloscope. Upon exposure to the pulsed laser, cobalt ferrite nanoparticles produced a high opto-acoustic (OA) wave. However when an a.c. magnetic field (50 Oe and 60 Hz) was applied, there was an attenuation in opto-acoustic (OA) emission, which suggests that there was an acoustic emission excited by the a.c. magnetic field, producing interference with the opto-acoustic emission. Furthermore when CSMENs were placed in the measurement cuvette with DI water, the intensity of the OA peaks decreased substantially, revealing that the barium titanate shell significantly absorbed the opto-acoustic wave emitted by the cobalt ferrite core. This variance in the propagation of the acoustic wave was due to the acoustic impedance difference between the two materials. Because the barium titanate shell is of a single crystalline nature as shown in Fig. 5, it will affect and change the potential at the surface. Figure 7(a) shows the opto-acoustic emission peak pulse time duration of 2.55 μs and intensity of 0.241 mV/mJ for cobalt ferrite nanoparticles, which decreased to 0.207 mV/mJ with 2.557 μs as the peak pulse duration, when an a.c. magnetic field was applied. When CSMENs were measured, the PA peak further reduced to 0.687 mV/mJ and the peak pulse duration was delayed to 2.585 μs due to the BT coating. In general, the basic mechanism of magnetoelastic emission is the generation of elastic pulses driven by magnetostriction. While there will also be emissions due to irreversible displacements of 90° (71° and 109°) domain walls, this is not significant in nanoparticles due to the large anisotropy energy involved. These pulses carry information regarding changes in the magneto-elastic state of a ferromagnet, as well as the elastic impedance involved in the transmitting body. Therefore, the magnetoelastic emission effect is sensitive to both the magnetic and elastic properties of a ferromagnet. Magnetoelastic vibrations in a ferromagnet occur when the applied magnetic field is time varying in nature. Pulses of acoustic emission generated in the process of cyclic magnetization are measured in most cases using piezoelectric transducers such as lead zirconate titanate (PZT’s) or by an ultra-high-frequency laser Doppler vibrometer, as discussed in literature^37^,^38^,^39^,^40^. This experiment corroborates that exciting the core of CSMEN with optical frequency results in opto-acoustic emissions and that these elastic waves can be fully absorbed by the BT shell. Via optimization of the BT layer thickness one can tailor the PA emission of the CSMEN for given pulse duration cycles.

#### Millisecond electric pulse generation by CSMEN

Calculation of the magnetically controlled surface potential change of nanoparticles, due to the absorption of the pressure wave by the BaTiO_3_ shell created by the magnetostriction of the core in an a.c. magnetic field is conducted and experimentally evaluated to demonstrate the millisecond electric pulse generation capability of CSMEN. Assume that the core and shell interface has a strong mechanical bonding such that the entire strain at the core is transferred to the shell. The surface potential generated by the shell will follow the magnetostriction frequency. The localized surface potential generated by the CSMEN is of a few tens of milli-volts and an 8.33 msec pulse when excited by a remotely applied a.c. magnetic field. The initial surface charge of BaTiO_3_ under no excitation conditions (i.e., spontaneous polarization) is confirmed through the zeta potential measurement of magnitude −24 mV for CSMENs with CFO:BT (60:40) as shown in Fig. 4. The electric field E_j_ generated by the CSMEN can be calculated using the following constitutive equation^41^


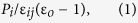


where, permittivity (ε_ij_) is a 2nd rank tensor of size [3 × 3] and **ε**_**0**_ is the vacuum permittivity and is equal to 8.854 × 10^−12 ^F/m.

For BaTiO_3_^42^,^43^,^44^, 
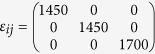
.

(P_i_) is a 1^st^ rank Polarization of size (3 × 1) in (Coulomb/m^2^) that can be expressed as^44^,^45^


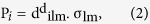


where, d_ilm_ is the 3^rd^ rank direct piezoelectric coefficient of size (3 × 6) with units of (Coulomb/N). Condensed Voigt notation is adopted in this and the following matrix expression.

For BaTiO_3_, the d_ilm_ is expressed as^42^,^43^,^44^


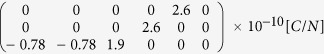


σ_lm_ is the 2^nd^ rank stress vector of size (6 × 1) N/m^2^ and can be related to 2^nd^ rank unit-less strain vector (x_jk_) of size (6 × 1). The relation can be written as^43^,^44^


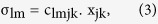


where, c_lmjk_ is the fourth rank stiffness/elastic matrix of size (6 × 6) in [N/m^2^],

For barium titanate the stiffness/elastic matrix^42^,^43^,^44^ has the form


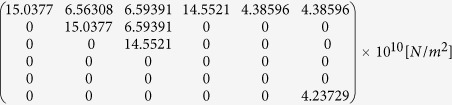


To calculate the magnetostriction of the cobalt ferrite core, we express the exciting a.c. magnetic field as;


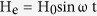


where, H_0_=50 Oe, ω = 2πf and f = 60 Hz have been used in this calculation. Magnetostriction or strain at a given instant (λ_i_) in m/A experienced by the core of CSMEN can be expressed as^45^,^46^,^47^,^48^,^49^,^50^


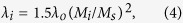


where, the magnetostriction coefficient of Cobalt Ferrite (λo) is adopted based on reported values of −1.48*10^−9^ m/A^45^,^49^,^50^. The Saturated magnetization (M_S_) was obtained from the hysteresis curve in Fig. 6 as 1.2*10^−2^ A/m.

Magnetization at any instant (M_i_) can be evaluated from magnetization versus applied field data in Fig. 6. The value of applied field at any instant is equal to H_0_* sinωt and magnetization (M_i_) is the value corresponding to that particular field in the ferromagnetic hysteresis loop in Fig. 6.

The maximum strain in a given direction can be calculated using^51^,^52^,


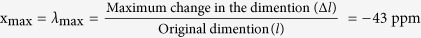
. This periodic strain change in the magnetostrictive core creates the elastic waves^50^.

In summary the magnetoelectric emission is a measure of strain mediated conversion of the magnetic field into the CSMEN’s polarization change via strain transfer from magnetostrictive core to the piezoelectric shell. The direct piezoelectric effect of barium titanate converts stress imposed by the core to a change in the surface potential of the CSMEN. This coupled phenomenon can be expressed by a magnetoelectric coefficient of 2^nd^ rank tensor of size (3 × 3)^44^:

The change in polarization ΔP_i_ = magnetoelectric coefficient (α_in_)* change in magnetic field (ΔH_n_), i.e.,


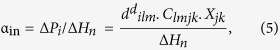


**Figure 8:**
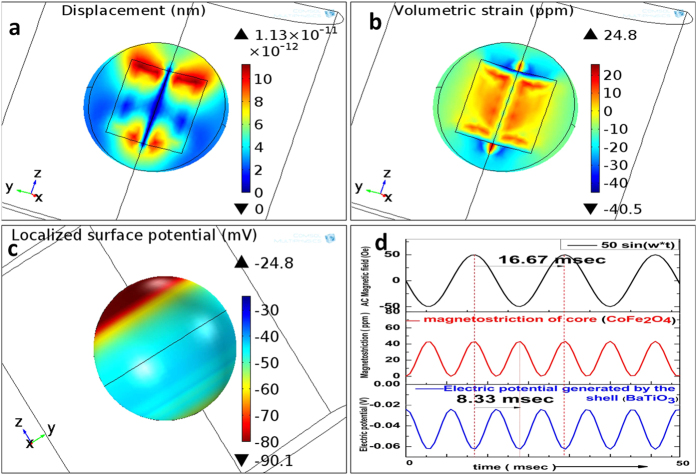
CSMEN behaviour in an a.c. magnetic field. (**a**) strain in the CoFe_2_O_4_ core is transferred to the BaTiO_3_ shell when exposed to alternating magnetic field; **(b)** Displacement in the shell caused by the stress transferred from the core and the displacement is periodic; **(c)** Localized electric potential created in the shell and; **(d)** The magnetostrictive pulse creates an electric pulse of 8.33 milliseconds (approx) corresponding to the excitation of an a.c. magnetic field of frequency 60 Hz.

The maximum magnetostriction coefficient achieved in the CSMEN when applying the given a.c. magnetic field excitation is α_max_ = 10.25 μV/nm*Oe,

As obtained from finite element (COMSOL Multiphysics 4.4) simulations (shown in Fig. 8), when the core-shell magnetoelectric nanoparticles are exposed to an alternating magnetic field, (a) the CoFe_2_O_4_ core experiences strain which emits an elastic wave. (b) The pressure wave is transferred to the BaTiO_3_ shell, and (c) the localized electric potential created in the shell. The magnetostrictive pulse creates an electric pulse of 8.33 milliseconds duration corresponding to that of an excitation magnetic field frequency of 60 Hz. The magneto-elasto-electroporation (MEEP) effect opens the nanopore on the cell membrane, and the movement of drug loaded nanoparticles can then be controlled by magnetic field application direction to penetrate through this electrically opened nanopore by the magnetic moment of the nanoparticle i.e., attraction toward the magnetic field.

When CSMENs are within few nanometer/sub-micrometer distance from biological cells, e.g., HEP2 cells, the continuous fluctuation of surface potential in the form of electric pulses exerts highly localized pulses in the mV/nm intensity range, which results in the cell’s external electric field change (U_ext_) in the vicinity of the cell membrane. As a result, the interaction of the electric pulse generated by CSMEN under the influence of an a.c. magnetic field induces the repulsion and dislocation of the phospholipids of the cell membrane, which primarily results in the opening of the nanopores and a change in the transmembrane voltage of the cell. This transmembrane voltage change is influenced by the electric potential generated by the CSMEN and represents the difference between external (V_ext_) and internal voltage (V_int_) of cell i.e. (V_m_ = V_ext_ − V_int_) and can be expressed as^2^


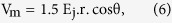


where, E_j_ represents the electric potential generated by the CSMEN, r is the radius of the HEP2 cells (approx. 15 μm) and θ is the angle between the CSMEN and the site of the cell membrane. Currently to achieve such a high localized potential (~mV/nm) throughout the large surface area of cell, tungsten nano-electrodes were used connected to a kV/cm generation source with millisecond to nanosecond pulse generating capability. Here we demonstrate that such fields can be generated and tailored to the biological system at the nanoscale by using CSMEN and remotely applied directional a.c. magnetic field. In addition the MEEP phenomena can be efficiently used to achieve controlled and targeted cell electroporation.

#### Magnetic moment of nanoparticles

The magnetic moment of a magnet is a property that determines the torque it will experience in an external magnetic field which is proportional to the forward movement velocity of particles due to attraction of particles towards magnet in high and low degree of freedom. The movement is time and frequency dependent and can define the time of CSMEN penetration into the biological cell.

Forward motion of the nanoparticles due to attraction force exerted by the magnets can be calculated by magnetization curve shown in Fig. 6. Ferromagnetic material such as CoFe_2_O_4_ nanoparticles or CSMEN multiferroic nanoparticles are complex physical object since both quantum and classical degrees of freedom have to be taken into account to describe their behaviour in external AC magnetic field. As discussed in literature^53^, the particle angular frequency (ω) and tensor of inertia represent the classical degrees of freedom of a nanoparticle. The tensor of inertia represented by I is considered for a spherical ferromagnetic nanoparticle. The quantum degrees of freedom are described by a macro-spin (S). S in the quasi-classical approximation that is defined as the ratio of the particle total magnetic moment to the gyro-magnetic ratio (γ), S = −Ms V α/γ, where *Ms* is the saturation magnetization, *α* is the unit magnetization vector and *V* is the particle volume. According to the quantum mechanical principle, the total momentum of the particle (J) is the sum of the mechanical angular momentum, L = I ω, and the total spin momentum (S), is conserved for an isolated nanoparticle like the example described in literature^53^.


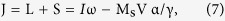


we have recorded this movement of CSMEN under influence of AC magnetic field using Fluorescence microscope bright field images and are shown in supplementary material- (Fig. S3).

### Biological Analysis of MEEP effect

#### Cytotoxicity Test

Human epithelial cell line Hep-2 (ATCC CCL-23) was used in this study. Hep2 cells were cultured in Eagle’s Minimum Essential Medium (EMEM) (ATCC,USA) supplemented by 10% of Fetal bovine serum (ATCC,USA). Cells were cultured in a 5% CO_2_ environment at 37 °C. Cytotoxicity of the BaTiO3 coated CFO nanoparticles was determined using MTS assay in 96 well plates. Cells were counted using a cell counter (Countess, Invitrogen) and approximately 1 × 10^5^ cells were seeded in each well. The cells were incubated overnight at 37 °C in a 5% CO_2_ environment. Different concentration (2 μg/ml, 10 μg/ml, 20 μg/ml, 50 μg/ml) of nanoparticles was added to each well and was further incubated for 24 hours. Cells with no nanoparticles added were used as a control. After 24 hours of incubation, the media was replaced with fresh media and 20 μl of MTS [3-(4,5-dimethylthiazol-2-yl)-5-(3-carboxymethoxyphenyl)-2-(4-sulfophenyl)-2H-tetrazolium] solution was added to each well and incubated for 4 hour at 37 °C. Absorbance at 490 nm was measured using Biotek Plate reader. Cytotoxicity was measured for concentrations up to 50 ug/ml. For all concentrations, measured percentage of viable cells compared to the control indicate cells continue to proliferate. This also supports that concentrations of the CSMENs used for transmembrane permeability studies are non cytotoxic. The results can be found in supplemental material (Fig. S4(a)) of this paper. Further studies were carried out to determine any combinatorial affects of nanoparticles and electromagnetic field on cell viability. The d.c. and a.c. magnetic field dependent and time dependent cytotoxicity graph and data can be found in the supplemental material (Fig. S4(b)), where it can be seen that no cytotoxicity is observed even while applying magnetic field for various time periods.

#### Transwell Experiment: Cell penetration by CSMEN analysis

**Figure 9:**
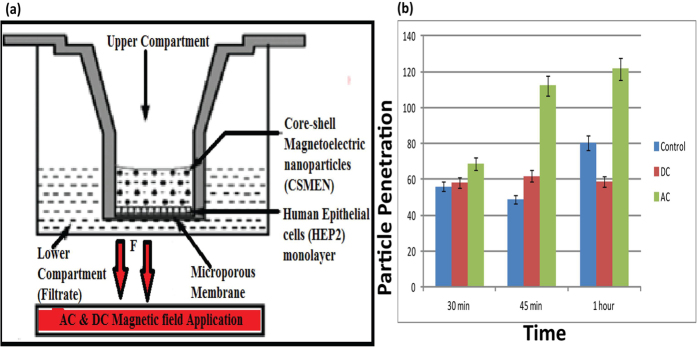
Schematic and results of the Transwell measurement. (**a**) Diagram of Transwell experiment; **(b)** Transwell graph shows increased filtrate intensity over time in presence of an a.c. Magnetic field whereas the fluorescence intensity of filtrate in case of a d.c. magnetic field and control remains minimal.

To analyze the penetration of CSMEN into HEP2 cells in the presence of an a.c. magnetic field, Transwell experiments were performed using the following procedure^54^. The penetration of nanoparticles into cells was evaluated using a polyethylene teraphthalate (PET)-coated control cell culture insert with a 1 micron pore diameter. Control inserts in 24 well plates were first seeded with HEP2 cells at a cell density of 1 × 10^5^ per inserts; then, 500 μl of phosphate buffer solution was added at the bottom of each well with control inserts. After the cells were grown to 100% confluence, the media was replaced with fresh media containing FITC conjugated CSMENs and was incubated for 30, 45 and 60 min. The FITC conjugation to CSMEN was confirmed using UV-Vis spectrophotometry measurements as shown in the supplementary material (Fig. S5). Cells without any CSMEN were used as a negative control. Cells with nanoparticles in the absence of an external magnetic field were used as a sham. The supernatant and the filtrate were collected and the fluorescence intensity at 490 nm was determined using BioTek micro plate reader. The schematics of the Transwell experiment under the influence of d.c. and a.c. magnetic fields are shown in Fig. 9(a). The Transwell graph in Fig. 9(b), shows increased filtrate intensity over time in the presence of an a.c. magnetic field, whereas the fluorescence intensity of the filtrate in the case of a d.c. magnetic field and control remains minimal. These data suggest that under an a.c. magnetic field, more CSMENs enter and pass through the cell due to the magneto-elasto-electroporation (MEEP) effect. However, in the case of a d.c. magnetic field, despite forward movement of the particles due to the Lorentz field effect, an absence of MEEP effect prevents the CSMEN from entering the cells.

## Discussion

The crystallographic characterizations of core-shell magnetoelectric nanoparticles conducted in this work have allowed us to conclude that the CSMENs fabricated are single crystal composites that consist of both CFO and BT single crystals with well-defined orientation correlations. While the PLD growth of the CFO on the BT substrate^55^ and CFO:BT pillar-matrix layered composite^56^ was reported, the in-plane and out-of-plane hetero-epitaxial growth relations have not been experimentally reported. As shown in Fig. 2 of our study, the thin BT layer followed the CFO morphology. PFM measurement has confirmed the single crystalline state of the barium titanate shell, as shown in Fig. 5, demonstrating that poling is not required because BT’s polarization states are apparently dictated by the termination layer and the negative surface potential of the CFO core. The conform growth of BaTiO_3_ single crystal layers on CFO crystal nanoparticles represents a new pathway for multiferroic composite fabrication and coupling especially in spinel-perovskites core-shell nanostructures, in which BT’s polarization states and the CFO’s spin states are evidently strongly coupled at their interface. The TEM image shown in Fig. 2 and the DLS measurement in Fig. 3 have confirmed that for a 60:40 composition ratio of CFO: BT, the size of the CSMEN is ~78.8 nm with a coating of 19–20 nm of the BT layer over a 50 nm diameter CFO core. The PFM measurements and ferromagnetic hysteresis curves shown in Figs 5 and 6 respectively, demonstrate distinctive and well-defined ferromagnetic and ferroelectric properties. The hydrothermal process of fabrication allows for BT crystalline formation at a substantially lower temperature that likely prevented phase diffusion, in comparison with conventional PLD fabrications, which are followed by sintering temperature exceeding 1200 °C^57^. The thickness of the hetero-epitaxial grown BT layer can be tailored to achieve the desired surface potential, by adjusting the ratio of the CFO: BT and by the choice of the sizes of the CFO cores. As shown in Fig. 4, the surface potential remains negative upon the growth of the BT layer on CFO nanoparticles and increases in magnitude as the layer thickness increases, until it reaches an optimal thickness; after that, the surface potential starts to decrease. The surface potential corresponds to a uniform and likely single domain polarization structure that is subsequently replaced by multi-domains or by polycrystalline formations, both of which can bring the negative surface potential to reduce to neutral, and diminishes the functionality of the nanoparticles. The acoustic measurement in Fig. 7 revealed the opto-acoustic and magneto-elastic property of cobalt ferrite nanoparticles and also verified the absorption of the elastic wave by the BaTiO_3_ single crystal shell. These experiments demonstrate exquisitely the magnetoelectric effect, through which an effective stress transfer from the core to the shell took place at a nanoscale, leading to a surface potential change under the excitation of an a.c. magnetic field.

**Figure 10:**
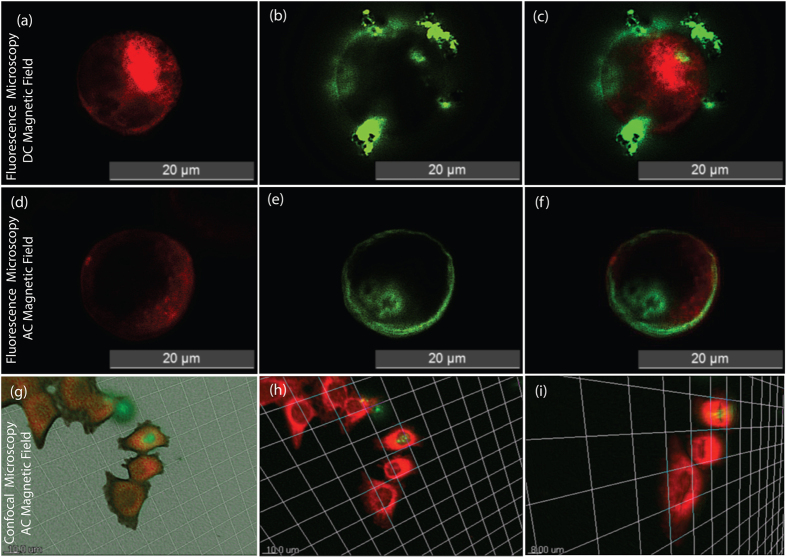
Fluorescence microscope images and confocal microscope z-stack images. Fluorescence microscope image of HEP2 cells interaction with CSMENs when exposed to a d.c. magnetic field and point of view imaged in, **(a)** fluorescent microscope red filter mode; **(b)** fluorescent microscope green filter mode; **(c)** Fig. 10(a,b) merged together. FITC loaded nanoparticles can be seen outside the HEP2 cells; **(d)** Fluorescence microscope image of HEP2 cells interaction with CSMENs when exposed to an a.c. magnetic field and point of view imaged in, **(e)** fluorescent microscope red filter mode; **(f)** Particles penetrated into the HEP2 cell and scatters the green fluorescence with cell membrane as a scattering boundary and can be observed in fluorescent microscope green filter mode; **(f**) Fig. 10(d,e) merged together. FITC loaded nanoparticles can be seen penetrated inside the HEP2 cells; **(g,h)** Confirmation of CSMEN penetration into HEP2 cells under influence of an a.c. magnetic field using confocal microscope z-stack images. The scale bar is 10 μm; and **(i)** Zoomed and inclined view confirmation of CSMEN penetration into HEP2 cells using confocal microscope z-stack images with scale bar as 8 μm.

As studied earlier^58^, the magnetoelectric CoFe_2_O_4_-BaTiO_3_ nanoparticles (size ~30 nm) were demonstrated as efficient carriers for high specificity drug delivery to eradicate ovarian cancer cells and were shown to be suitable for the externally controlled on-demand release of anti-HIV drugs^59^. A d.c. magnetic field was used for the targeted nanoparticles movement^57^ whereas d.c. and a.c. magnetic fields above certain threshold field levels were used for the on demand release of anti-HIV drugs; the later was found to be more efficient^58^,^59^. In this work we have designed experiments and obtained visual verification of CSMEN penetrating into a selective cell by opening nanopore on the cell membrane under excitation of an a.c. magnetic field via the electroporation proce\ss, as shown in Fig. 10. This is achieved by a combination of controlled transport to the surface of the targeted cell, followed by a deliberate penetration of the targeted cell driven by an a.c. magnetic field. The pulsation of the CSMEN surface potential at the second harmonic of the remotely applied a.c. magnetic field frequency as a result of the direct piezoelectric effect is also quantitatively evaluated using the finite element method. The induced surface potential change of ~−40 mV peak-to-peak for a CSMEN driven by a 50 Oe and 60 Hz a.c. magnetic field is shown to be significant in driving the penetration of the targeted cell membranes. The transferred magnetostrictive strain from the CFO core is extremely small (of the order of ~40 ppm), indicating that the electromechanical transduction is highly efficient. Transwell experiments have recorded the penetration of the nanoparticles, to a large extent from the HEP2 cells when subjected to an external a.c. magnetic field excitation, compared to a d.c. magnetic field (see Fig. 9). This proves that CSMEN under the influence of an a.c. magnetic field generates electric pulses. When these pulses interact with nearby cell, magneto-elasto-electroporation (MEEP) occurs primarily because of phospholipid dislocation on cell membrane which in-turn effect the transmembrane voltage of the cell.

To visually verify the MEEP phenomenon, a longitudinal penetration experiment and analysis was performed on human epithelial cells (HEP2). The HEP2 cells were seeded at a cell density of 1 × 10^5^ per well in 24-well plate. Fluorescein isothiocyanate (FITC) dye loaded on the silica-coated CSMENs (for confirmation of FITC loading on CSMEN, refer to the supplementary material (Fig. S5)) of 50 μg/ml concentration were then incubated with the cells, and different intensities of the a.c. and d.c. magnetic fields were applied for varying timeframes. The intensity of the d.c. field was varied from 50 Oe–200 Oe, and the intensity of the a.c. magnetic field was varied from 50–100 Oe with a frequency of 60 Hz. CSMENs were added to one end of the well, and the magnetic field was applied to the other end. The HEP2 cells were present in the space between the CSMEN and the applied a.c. magnetic field; thus, the MEEP phenomena occurred as the CSMENs in response to applied a.c. magnetic field creates nanopores on the cell membrane, hence allowing the nanoparticle to penetrate through the electrically opened nanopores towards applied magnetic field. The cytoplasm was stained with red cytoplasmic stain (CellMask) according to the manufacturer’s protocol; cells were then fixed using a fixative agent, attached to the glass slide and observed under a fluorescence microscope and confocal microscope. Fluorescence microscopy images and confocal microscopy z-stack images were obtained, and all the individual fluorescence microscopy images taken at the same spot in red and green filters were merged using the ImageJ software. In the presence of a d.c. magnetic field, CSMENs were observed to be outside the HEP2 cell membrane, as shown in Fig. 10(a–c). In the fluorescence microscopy images, the green fluorescence outside the cell periphery indicates that the particles did not penetrate the cell membrane under the influence of a d.c. magnetic field. Instead the CSMEN tended to accumulate outside as expected due to the lack of a continuous change in the surface potential of CSMEN which stems from the lack of oscillating field excitation. When HEP2 cells were targeted with CSMENs exposed to an a.c. magnetic field for various time periods, CSMENs loaded with FITC with green fluorescence emission were observed in the cell periphery, and a scattering of the green fluorescence was observed within the cell membrane, which behaved as the scattering boundary. This is shown in Fig. 10(d–f), indicating that the CSMEN penetrates into the HEP2 cells. These fluorescence images also indicate that these CSMENs can also be used for image resolution enhancement due to their electromagnetic field coupling enhancement. This penetration of CSMENs into the HEP2 cells under influence of an a.c. magnetic field was further confirmed using confocal microscopy z-stack images, as shown in Fig. 10(g–i). CSMEN penetration can be observed inside the HEP2 cells. Flutax software was used for the optical slicing of different focus planes on the confocal microscopy z-stack images and analysis. Nanoparticles were found to be present inside some of the slices of cells, as shown in Fig. 10(g–i), which clearly indicates that CSMENs have penetrated into the cells under the influence of an a.c. magnetic field.

We conclude that single crystalline BaTiO_3_-coated CoFe_2_O_4_ - CSMEN have the potential to be used primarily as nano-probes for bio-sensing, performing controlled and targeted electroporation and studying a cell’s electrical properties and ionic activities. The medical therapeutics field and the nanoscale bio-sensing field will have a strong impact because both can be performed simultaneously by elaborately exploring the MEEP effect.

## Methods

### Synthesis of BaTiO_3_ Coated CoFe_2_O_4_ Nanoparticles

The Core-Shell Magnetoelectric (CSMEN) nanoparticle composites were synthesized using hydrothermal methods. The CoFe_2_O_4_ nanoparticles used were obtained from commercial source (Alfa Aesar Inc.) Barium Carbonate (BaCO_3_) and Titanium Iso-propoxide (Ti(OCH(CH_3_)_2_)_4_) were mixed with citric acid in separate containers to obtain the Ba and Ti citrate solutions. To form uniform coating of BaTiO_3_ on CoFe_2_O_4_ nanoparticles, these citrates were then mixed with CoFe_2_O_4_ nanoparticles in Ethylene Glycol and heated at 100 °C to paralyze the solution. To stabilize the barium titanate shell and achieve intended crystalline characteristics, the mixture is dried and heated further at 800 °C for 8 hour in a low supply of oxygen to prevent oxidation of the ferromagnetic nanoparticles. Finally the dried powder was repeatedly washed using ethanol and de-ionized water and sonicated in ultrasound cleaner to obtain the final crystallized composites of BaTiO_3_ coated CoFe_2_O_4_ nanoparticles.

### Sample preparation for AFM and PFM measurements

PFM measurement on nanoparticles is very complex since we need a substrate with atomically smooth surface (where surface roughness is very low/lower that the particle size-in nanometers). Moreover particles must stick to the surface of the substrate and should be immobilized so that Voltage can be applied using PFM tip and tip can scan the nanoparticles at the same spot as it was on each scan. As shown in literature, to achieve this we use a Mica substrate and cleaved its surface multiple times (4–5 times) by using adhesive tape. With this process we got an atomically smooth surface. Then this cleaved mica substrate was carefully immersed in a mixture of (1:5) Poly-L-Lycin and DI water for 25 mins. This process will make the Mica substrate surface positively charges. Since nanoparticles have negative zeta/surface potential (discussed in earlier part), the nanoparticles stick to the surface of Mica and remain immobilized. Thus both AFM and PFM scanning can be done efficiently.

### Cytotoxicity Test

MTS assay was performed for cytotoxicity test. 2. Epithelial cell line Hep2 was used for the test. 3. Briefly, 10^5^ cells were seeded in each well in 96 well plate with 100 ul of culture media. After 24 hour, media was replaced with media containing the samples in different concentration. The concentration used were 2 ug/ml, 10 ug/ml, 20 ug/ml, 50 ug/ml, 100 ug/ml, 200 ug/ml, 500 ug/ml and 1 mg/ml. The cells with samples were incubated for 24 hour. 4. Then, the media was replaced with 100 ul of fresh media and 20 ul of MTS solution was added to each well. After incubating for 4 hour hour, absorbance at 490 nm was measured using Biotek Plate reader.

The MTS [3-(4,5-dimethylthiazol-2-yl)-5-(3-carboxymethoxyphenyl)-2-(4-sulfophenyl)-2H-tetrazolium] tetrazolium compound is bioreduced by metabolically active cells in to a colored formazan product that is soluble in tissue culture medium. This conversion is accomplished by NADPH or NADH produced by dehydrogenase enzymes in metabolically active cells. The data is presented in Supplementary material- (Fig. S3) of this article.

### FITC conjugation on Si Coated CSMEN

FITC was first conjugated to APTES. Typically, FITC (2 mg) was dissolved in 0.1 M APTES in ethanol. The solution was stirred in dark for 24 hour. FITC-APTES (5 ml) solution was then added to silica coated particles (10 mg) and was stirred vigorously for 1 hour. The solution was then incubated for 24 hour at 40 °C. The resulting solution was washed repeatedly by ethanol to remove un-conjugated FITC. The FITC loading on silica coated CSMEN was confirmed using spectrophotometer measurement and results are shown in supplementary material (Fig. S4).

## Additional Information

**How to cite this article**: Betal, S. *et al.* Magneto-elasto-electroporation (MEEP): *In-vitro* visualization and numerical characteristics. *Sci. Rep.* **6**, 32019; doi: 10.1038/srep32019 (2016).

## Supplementary Material

Supplementary Information

## Figures and Tables

**Figure 1:**
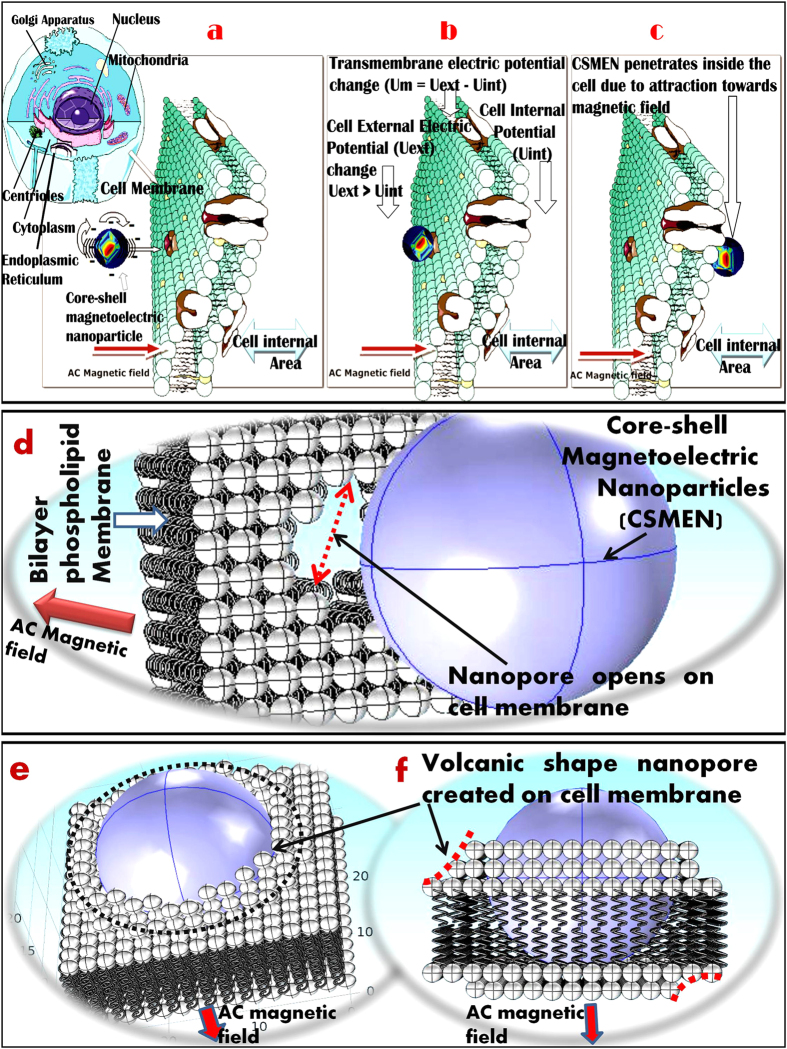
Schematics of the MEEP mechanism. **(a)** The area of the cell membrane with the CSMEN particles at the vicinity under the influence of an a.c. magnetic field experiences anisotropic strain in the core (CoFe_2_O_4_) and high localized surface potential change on the shell (BaTiO_3_); **(b)** On the other side of the cell is an electro magnet which attracts the CSMEN towards itself. Within a few nanometer distances from cell membrane, a CSMEN changes the cell’s external potential U_ext_ as the magnetic field is turned on, resulting in nanopore opening on cell membrane; **(c)** CSMEN penetrates through the electrically opened nanopore towards the magnetic field due to Lorentz force; **(d)** Schematics of pore opening phenomena on cell membranes due to the CSMEN as a result of displacement of phospholipids; **(e,f)** Various plane view of the volcanic shaped nanopore on bilayer phospholipid membrane from which particle penetrates into the cell and continue to move towards the magnetic field.
