# Supplementary material for: Magneto-elasto-electroporation (MEEP): In-vitro visualization and numerical characteristics
Source: Sci Rep. 2016 Aug 26;6:32019. doi: 10.1038/srep32019 (PMC4999954; doi:10.1038/srep32019)
Supplement: Supplementary Information [file srep32019-s1.pdf]

Magneto-elasto-electroporation (MEEP): In-vitro visualization and numerical characteristics

Soutik Betal*[a], Binita Shrestha[b], Moumita Dutta[a], Luiz F. Cotica[d], Edward Khachatryan[c], Kelly Nash[c], Liang Tang[b], Amar S. Bhalla[a], and Ruyan Guo[a]

[a] Department of Electrical and Computer Engineering, University of Texas at San Antonio, San Antonio, TX 78249, USA. [b] Department of Biomedical Engineering, University of Texas at San Antonio, San Antonio, TX 78249, USA, [c] Department of Physics and Astronomy, University of Texas at San Antonio, San Antonio, TX 78249, USA [d] Department of Physics, State University of Maringá, Maringá, PR – 87020-900, Brazil.

Supplemental Material

1. Atomic Force Microscopy measurements:

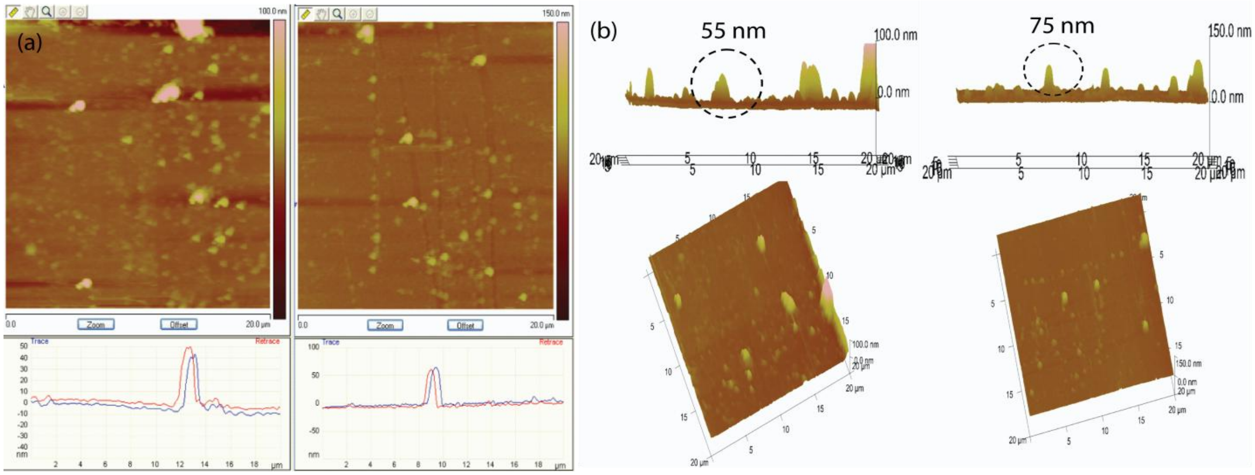

***Figure S1: (a) AFM scanning mode image-** shows the scanning of cobalt ferrite nanoparticles, data scale of the first image is (-50 to 50nm) and size in the 1st image is about 50-55 nm whereas the second image of CSMEN, data scale (-100 to 100nm) shows the size of CSMEN as 75-79nm; **(b) AFM Scanning** topography image shows the size and morphology*

2. Magnetic Force Microscopy (MFM) measurements

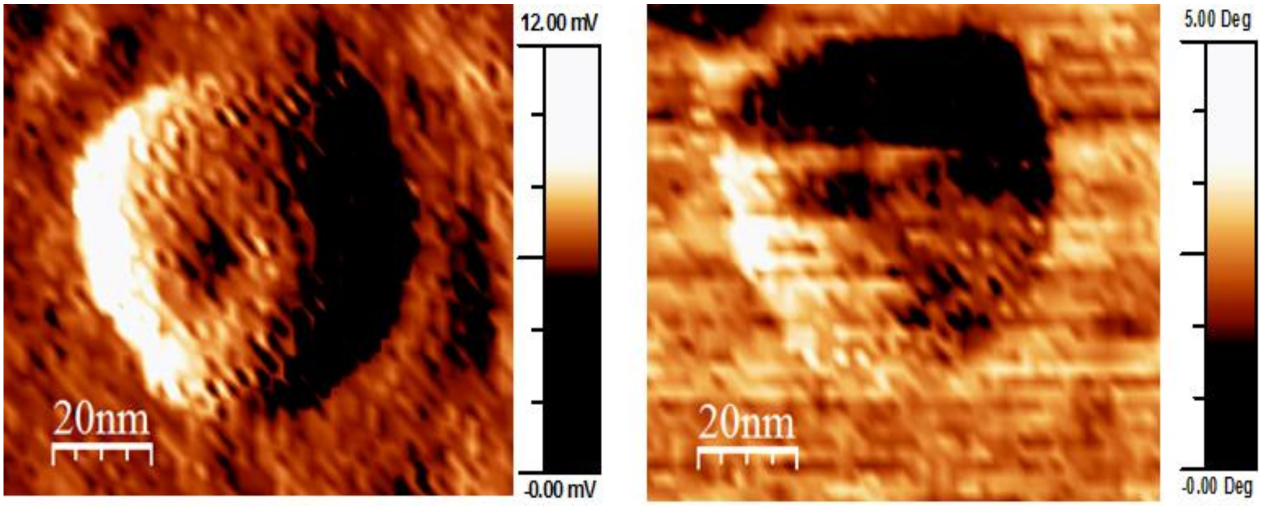

Figure S2: Magnetic Force Microscopic Images over an area of 100nmX100nm: (a) Amplitude (b) Phase. The amplitude and phase Images represent the amount of shift brought about to the intial amplitude and phase to which the tip was tuned to, by the magnetic interaction between the magnetic domains of the particle with that of the magnetized tip.While the darker regions signify attraction, the brighter regions is suggestive of repulsion.

3. Movement of CSMEN under influence of ac magnetic field

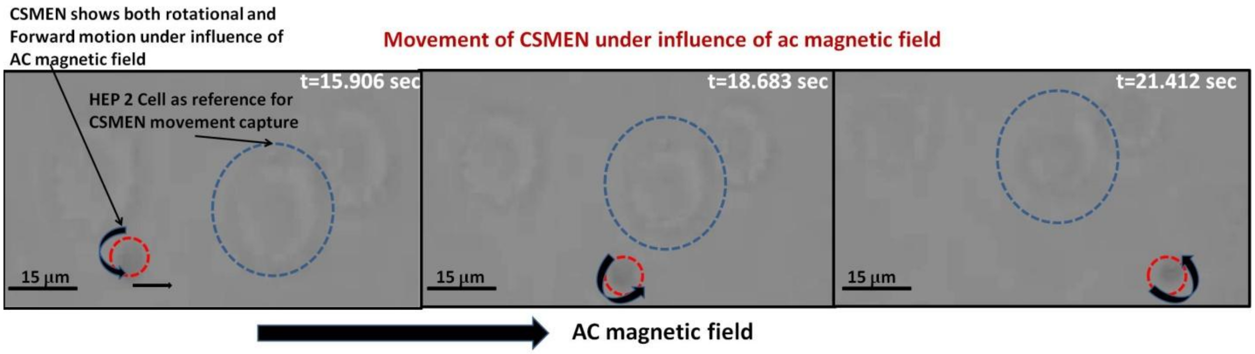

Figure S3: Time dependent movement capture of CSMEN under influence of ac magnetic field: CSMEN shows both rotational and forward motion under influence of AC magnetic field as analyzed keeping a HEP 2 Cell as reference.

4. Cyto-toxicity Test

1. MTS assay was performed for cytotoxicity test. 2. Epithelial cell line Hep2 was used for the test. 3. Briefly, 10^5 cells were seeded in each well in 96 well plate with 100 ul of culture media. After 24 hour, media was replaced with media containing the samples in different concentration. The concentration used were 2ug/ml, 10 ug/ml,20 ug/ml, 50 ug/ml, 100 ug/ml, 200 ug/ml, 500ug/ml and 1mg/ml. The cells with samples were incubated for 24 hour. 4. Then, the media was replaced with 100 ul of fresh media and 20 ul of MTS solution was added to each well. After incubating for 4 hour hour, absorbance at 490 nm was measured using Biotek Plate reader.

The MTS [3-(4,5-dimethylthiazol-2-yl)-5-(3-carboxymethoxyphenyl)-2-(4-sulfophenyl)-2H-tetrazolium] tetrazolium compound is bioreduced by metabolically active cells in to a colored formazan product that is soluble in tissue culture medium. This conversion is accomplished by NADPH or NADH produced by dehydrogenase enzymes in metabolically active cells. Cell viability test on time dependent d.c. and a.c. magnetic field application in presence of cells and CSMEN was also measured with concentration of 50 ug/ml. The cytotoxicity is always in control and hence depicts the safe usage of CSMEN for biomedical applications. cell viability more than control i.e. the number of live cells counted after each experiment shows results higher than what needed for non cytotoxicity confirmation.

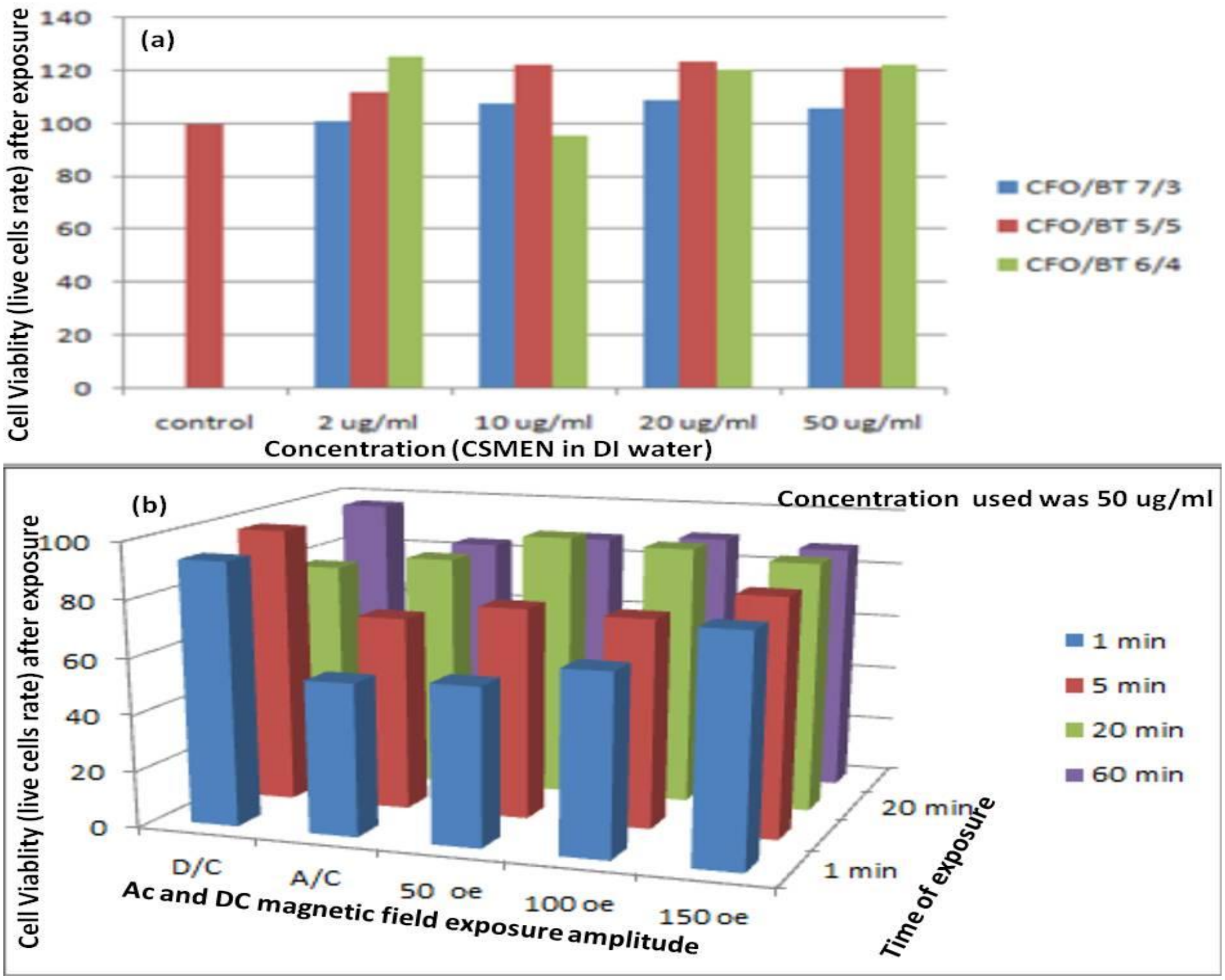

Figure S4: Cytotoxicity test Data: (a) MTS assay was performed for different composition of CSMEN, The graph shows the rate of live cells counted after the exposure of cells with CSMENs, (b) Time and magnetic field intensity dependent Cytotoxicity test- The graph shows the rate of live cells counted after the exposure of cells with CSMENs in AC & DC field with 50μg/ml concentration.

5. UV vis spectrophotometry results for FITC coating confirm on silica coated CSMEN

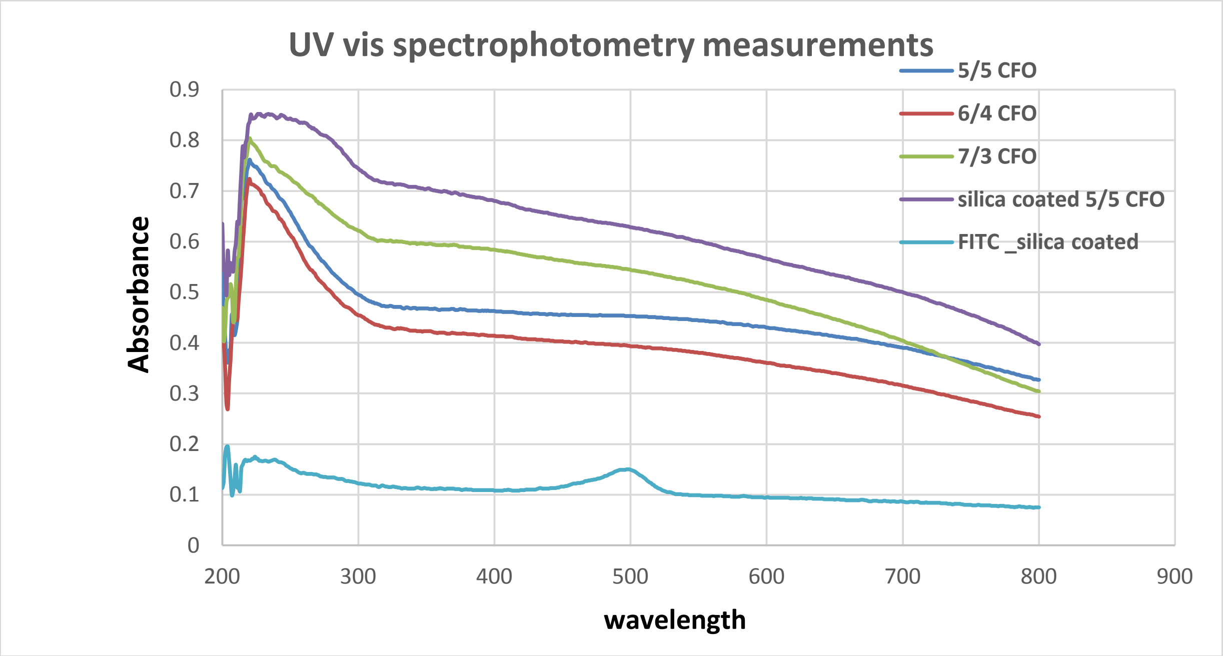

Figure S5: UV-vis spectrophotometry measurements for FITC coating confirmation on silica coated CSMEN since peak can be observed at 520 nm which is for green fluorescence emission of FITC

6. Supporting TEM image and Diffraction pattern

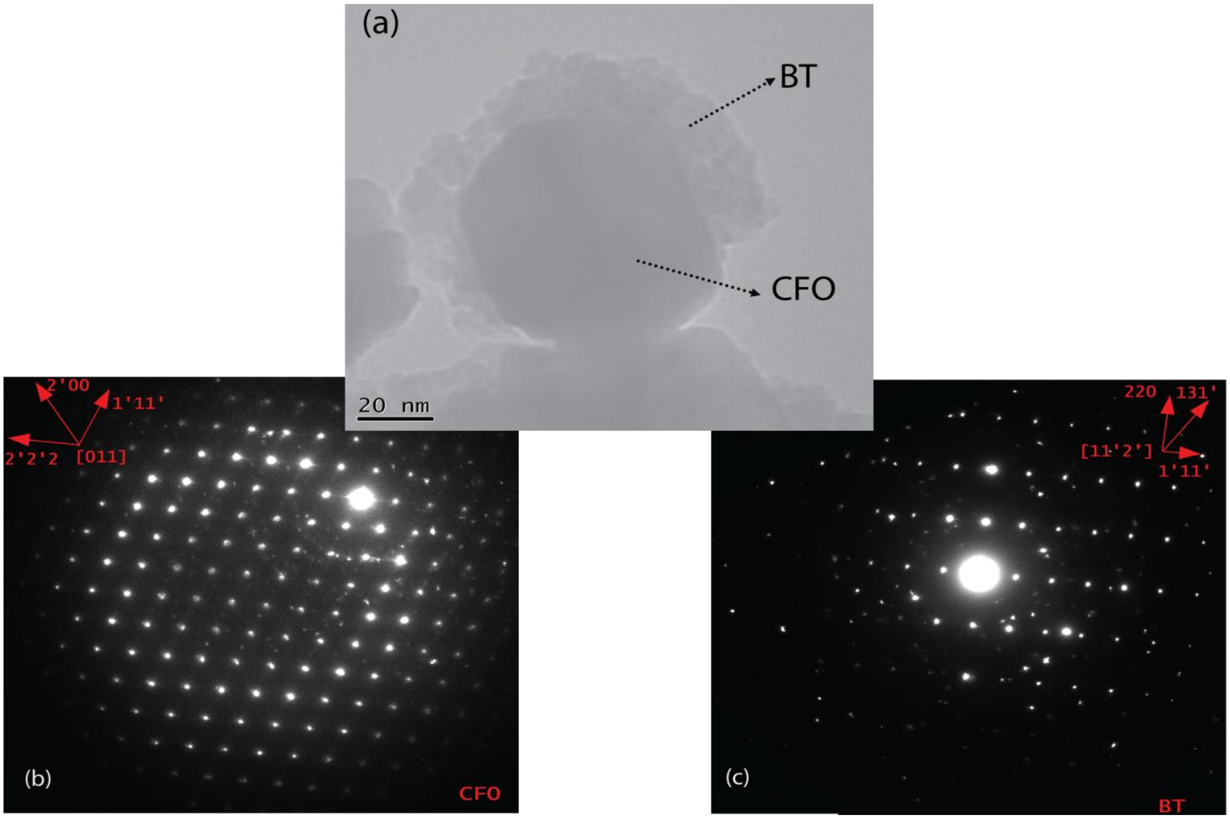

Figure S6:(a) Transmission electron microscopy image showing core-shell structure. Diffraction Pattern of (b) Cobalt Ferrite, Zone Axis [011] and (c) CSMEN with zone axis of single crystalline Barium Titanate as [11'2']
